# Setting Up an Ambulatory GI Endoscopy Suite in the USA—Anesthesia and Sedation Challenges

**DOI:** 10.3390/jcm13154335

**Published:** 2024-07-25

**Authors:** Basavana Goudra

**Affiliations:** Jefferson Surgical Center Endoscopy, Department of Anesthesiology, Sidney Kimmel Medical College, Jefferson Health, 111 S 11th Street, #8280, Philadelphia, PA 19107, USA; basavana.goudra@jefferson.edu

**Keywords:** GI endoscopy, out patient, propofol, sedation, organization, administration, ambulatory

## Abstract

Gastrointestinal endoscopy units, both freestanding and associated with ambulatory surgical centers, are on the increase, and the trend is likely to continue. The concept is relatively new, and there are insufficient guidelines and a general dearth of information for prospective planners and physicians. Debate continues in areas such as the selection of patients, appropriateness of procedures, and access to tertiary care. Leaders often scramble to address both critical and non-critical issues, often after the center has opened to the public. They often encounter issues which were not anticipated. In this review, we have provided comprehensive and concise information on the various aspects of starting and running an endoscopy unit. Some of the areas considered are referral and recruitment systems, determination of the need and site selection, layout and regulations, aspects related to drugs, equipment, medical emergencies, and emergency room transfers, discharge criteria, post-discharge follow-up, and finally, we have addressed issues related to avoiding and managing cancelations. It is assumed that a majority of the procedures are performed with predominantly propofol-induced deep sedation.

## 1. Introduction

Over the centuries, the medical profession has worked tirelessly to prolong human life and mitigate ill-health-related discomfort. One of the practical ways of accomplishing these goals is the use of screening tools. They have emerged as effective instruments for combating cancer (a modern epidemic), mainly by early detection. Considering the cost of surgery and chemotherapy, employing extensive screening measures saves both money and time. Gastrointestinal (GI) endoscopic procedures are at the forefront of such cancer-mitigating measures. These include colonoscopy and endoscopic ultrasound. Although extremely effective, the logistics of implementation are, nonetheless, a challenge. The aging population and changing guidelines have placed immense pressure on the service providers too. To be effective at a population level, such facilities must be accessible and cost-effective.

In this segment of the Special Issue, we have attempted to provide comprehensive knowledge which will assist both health care administrators and anesthesia/sedation providers in planning and managing a freestanding endoscopy unit.

## 2. Scale of Practice

Procedures performed in an ambulatory GI endoscopy suite depend on the location and the type of support the facility maintains. Such support includes their ability to transfer to another facility in case of an emergency and the level of expertise of the staff—medical, technical, and nursing. In general, depending on the center, the following procedures are considered appropriate for an ambulatory GI endoscopy suite.

Colonoscopy.
Esophagogastroduodenoscopy.
Endoscopic retrograde cholangiopancreatography (selected).
Sigmoidoscopy.
Selected single balloon and double balloon enteroscopy, both anterograde and retrograde.
Endoscopic ultrasound.
Therapeutic endoscopy such as endoscopic mucosal dissection/resection, peroral endoscopic myotomy.
Endoscopic mucosal resection (selected).

## 3. Patient Selection and Enlistment

Outpatient GI endoscopy procedures can be performed at various sites. However, a large proportion of these procedures are currently carried out in hospital-affiliated outpatient departments and ambulatory surgery centers (ASCs). In the USA, complexity of the procedure and the degree of patients’ comorbidity often dictate the site selection. Within the category of hospital outpatient departments, the presence of a conduit (such as a bridge or a tunnel) connecting the main hospital allows immediate ED transfer and allows greater flexibility in both patient and procedure selection.

Appropriate patient selection and enlistment is critical to the success of any ambulatory GI endoscopy suite. An effective and sensible screening and selection should ideally eliminate any cancelations or need for transfer to another facility. In the last decade, there has been a trend of performing most procedures in an ambulatory setting. Studies have demonstrated the safety of such an approach. By using procedure-specific, propensity-score-matched samples, and multilevel logistic regressions adjusting for patient, procedure, and facility characteristics, Meng-Yun Lin et al demonstrated that fewer patients treated in ASCs required unanticipated admission. In their study, the relative risk of having 7-day hospital visits after a screening colonoscopy performed in ASCs was 0.88 compared with hospital outpatient departments [1]. Nevertheless, efficient planning and execution are important to achieve such a high degree of safety, along with minimum complication and admission rates. Due to the inherent limitations of performing GI endoscopy procedures in an ASC, criteria need to be established and rigorously followed, both in terms of patient and procedure selection. As more information becomes available, these criteria and their implementation should be revisited and appropriately revised. Patient outcome and satisfaction are clearly important considerations. 

The patient pool for stand-alone, fully self-contained GI endoscopy centers comes from various sources. These include the following:

**Self-referral or referral by participating (in the health system) or by non-participating doctors.**

This is probably the single largest pool, and the numbers depend on the efforts made to encourage patients’ participation. Providing an educational handout that prompts patients to discuss colonoscopy with their physician is known to increase referral [2]. With an increased awareness of colonic cancer, many patients contact providers (hospitals and physicians) online or via telephone and schedule their own colonoscopy. Other interventions known to improve screening colonoscopy uptake are health literacy training aimed at referring physicians, monitoring patient compliance using a feedback loop, and tracking the screening of eligible patients [3]. Most evidence confirms that a screening colonoscopy reduces colon cancer. Doubeni et al. demonstrated that the screening colonoscopy was associated with a 67% reduction in the risk of death from any colorectal cancer [4]. Bretthauer et al. came to similar conclusions [5]. The additional benefits of colonoscopy (in comparison to sigmoidoscopy alone) are, however, small [6]. 

Blood- and stool-based testing improves the follow-up colonoscopy, especially in medically underserved populations [7]. Other approaches are attempted too. Beverly B. Green et al. (2019) studied the impact of financial incentives on colonoscopy uptake. They did not find any significant increase in colorectal screening between those offered financial incentive versus those who were not, although fecal immunochemical test uptake was higher in the incentivized group [8]. Apart from the screening colonoscopy, patients might self-refer to a hospital or a gastroenterologist for other symptoms/diagnosis. These include acid reflux symptoms, abdominal pain, change in bowel habits such as constipation, per-rectal bleeding, difficulty or painful swallowing, unexplained weight loss, and persistent nausea/vomiting. This might be an appropriate time to discuss a screening colonoscopy. A family member being diagnosed with colonic or other GI cancer (stomach, pancreas, etc.) is another reason for referral.

**Referral from a family physician or another physician to a gastroenterologist.**

Another pool of patients presenting for both routine procedures such as the screening colonoscopy and advanced endoscopic procedures are referrals from other physicians and gastroenterologists. This is especially true in major tertiary centers where better expertise may be available.

Nonetheless, primary care physicians could play an important role in filling the shortage of qualified gastroenterologists, although such a role is largely limited to screening colonoscopies [9]. Many surgeons and other specialists trained in endoscopy perform a significant number of endoscopic procedures including screening colonoscopy. When performed by other endoscopists (other than gastroenterologists), adenoma detection rates, cecal intubation rates, polypectomy rates, flat polyp detection rates, carcinoma detection rates, sedation rates, and complication rates were similar [10].

**Repeat patients.**

The third group includes those who had prior colonoscopy and require a repeat procedure at an appropriate time depending on the findings at the previous colonoscopy and history. Generally, these patients are sent a letter to remind them of their procedure time and followed-up further by the appropriate team. A small number includes those who failed initial colonoscopy for reasons such as poor bowel preparation and sedation/anesthesia issues. 

## 4. Determining the Need and Site Election

There are many reasons for setting up endoscopy ambulatory surgery centers (EASCs). These centers are considered more efficient than hospital-based locations, less costly to taxpayers, and provide comparable safety [11]. As alluded to earlier, there is a demand for endoscopic procedures created by an aging population. The current guidelines recommending the first screening colonoscopy at the age of 45 years (through 75 years) will increase the need for endoscopy centers. As a result, local population dynamics and demography will determine the need for EASCs at any location. Separately, there is an increase in the prevalence of gastrointestinal disorders; these include gastrointestinal cancers, gastroesophageal reflux disease (GERD), inflammatory bowel disease, and gastrointestinal bleeding, and such a trend is seen globally [12]. Until recently, GERD was mainly seen as a problem of middle-aged and older people. However, the number of younger patients experiencing such symptoms has increased significantly. Fortunately, the prevalence seems to decrease with education and income level [13]. Surprisingly, the prevalence of GERD is higher among ex-smokers than current smokers and non-smokers. The consumption of acid suppressants such as proton pump inhibitors has increased in all age groups with the greatest increase seen in the group 30–39 years [14]. 

New GI-endoscopy-driven ASCs are much needed, and 30 such centers were opened in 2023 in US alone [15]. Apart from the need, factors such as financing and equipment/personnel utilization are to be taken into consideration. Some centers are private-equity-backed, and their participation in the investment and consolidation of health care should be appreciated and recognized [16]. It is said that about 18 months are needed to plan and build an ambulatory surgery center [17]. The following need to be considered while designing an ASC. At a very basic level, these centers should

1. Facilitate the performance of a mixture of high-revenue and high-volume cases. Clearly, GI endoscopy cases assist in increasing the volume of any ASC.
2. Understand and keep in alignment with market direction.
3. Follow the lean principles and operational flow and how these should support each other.

As David S. Stokesberry, MD stated in his thought-provoking paper “Ambulatory surgery centers 101: What new GIs need to know” published in *The New Gastroenterologist*, providing superior quality care is paramount. In this direction, each center must enter into an agreement with Medicare and meet its certification requirements, which are similar to those required for hospital outpatient departments [18]. The distinctions are mainly related to patient flows, patient selection, and the regulations. A “freestanding” surgery center falls under the classification of a hospital-based outpatient department facility if it is within a 35-mile radius of the hospital and regulated by the same financial and administrative contracts. Likewise, such a facility can be operated by a hospital, while maintaining an ASC status, if it is financially and administratively independent with its own Medicare agreement [19]. There are differences in terms of renumeration for similar procedures performed in these two locations, which are important savings for Medicare. For some procedures, it may be financially beneficial for the provider to perform at an ASC. In fact, it is stated that for two primary procedures (colonoscopies and endoscopies) performed at ASC, Medicare payments exceeded production costs [20]. 

At the Jefferson Surgical Center Endoscopy in Philadelphia, administratively a part of the Jefferson health system, the following criteria are used in selecting patients at various phases of scheduling (Table 1, Table 2 and Table 3). 

Table 1 is our direct access colonoscopy and procedure screening form, typically filled by schedulers before listing patients employing an open access (also called direct access) model. These are typically healthy patients wishing to undergo a colonoscopy because of their age or strong family history. They can bypass the gastroenterologist’s office visit, thereby avoiding additional expenses. The Society for Gastrointestinal Endoscopy (ASGE) Standards of Practice Committee has issued certain guidelines to referring physicians who wish to send their patients via this route [21]. Those found inappropriate for direct access are appropriately reviewed by a registered nurse, delayed, or sent for additional consultation. Pre-admission testing centers are typically staffed by nurses experienced in this area. The table also contains information on the bowel preparation for colonoscopy. In addition to the screening and scheduling described above, all patients receive a call from a specialized endoscopy nurse 7 days before the scheduled date to discuss any issues that might require scheduling. These could be related to a new diagnosis such as new stroke, MI, or procedures like coronary stents. 

It is important to avoid pitfalls such as inappropriate referrals, communication errors, and inadequately prepared or informed patients. Nevertheless, some of these recommendations might not be practical and might hinder patients from availing themselves of this valuable service. Sending invitations to eligible patients (for open access colonoscopy) via respective health care electronic medical records might boost open access participation; nevertheless, such an effort could only recruit 3.8% of 146,112 pts who received letters. Clearly, other methods are required to educate and encourage the eligible population [22]. Direct access colonoscopy may also be performed in primary care (as opposite to secondary care) with relative ease and success [23]. The safety and polyp detection rates are comparable between direct access and conventional colonoscopy. 

Table 2 contains the list of procedures considered appropriate for Jefferson Surgical Center Endoscopy. This list might be too restrictive for a center that has a bridge or tunnel to a hospital with emergency room admission facilities. Such a facility has almost no limitations in terms of procedures that can be performed. Contrarily, a freestanding facility in a relatively remote location needs to be even more restrictive. At Jefferson Surgical Center Endoscopy, we have an ambulance (Jeffstat) which is always positioned at the center and can transfer patients to our hospital (located across the street) in less than 10 min.

Table 3A,B list the criteria for patient appropriateness from the anesthesiology stand point. These criteria depend on the local expertise available and the ease of transfer to another facility in case of emergency and need for admission.

## 5. Floor Layout and Regulations

**Figure 1:**
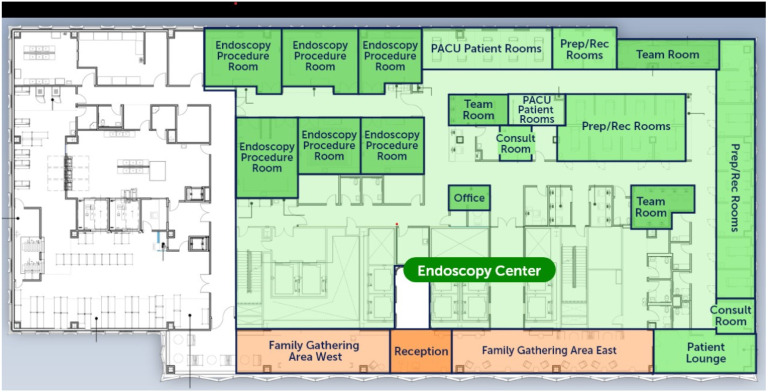
Floor plan of the endoscopy unit at Jefferson Surgical Center Endoscopy, Philadelphia, PA, USA.

Figure 1 is an example of an endoscopy suite design of a major university-hospital-affiliated freestanding endoscopy center, Jefferson Surgical Center Endoscopy. The center opened in May 2024. 

Conflicts might exist between the needs of various stakeholders, such as the patient, anesthesia providers, endoscopist, and nursing staff, and the mandates coming from the governing bodies. An example is the suitability of performing a specific procedure or group of procedures. While the various stakeholders might see it as appropriate, the regulatory bodies might not allow it. These bodies include (1) the State Department of Health—Licensing Division, (2) Medicare, and (3) the state fire marshal [24].

Medicare Claims Processing Manual Chapter 14 of the Centers for Medicare and Medicaid Services (CMS) manual [25] provides information related to payment under Part B for services provided by an ambulatory surgical center. The manual defines ASC as a distinct entity that operates exclusively for the purpose of furnishing outpatient surgical services to patients. An ASC could be independent or operated by a hospital; 42 CFR 416.25-49 discusses the conditions for coverage for ASCs.

In Pennsylvania (USA), the Department of Health, Division of acute and ambulatory care is responsible for the licensing and oversight of Pennsylvania’s ambulatory surgical facilities [26]. In turn, the states’ requirements are imposed by the Centers for Medicare and Medicaid Services.

## 6. Drugs, Equipment, Medical Emergencies, and Emergency Room Transfers

Table 4 lists the drugs that are made available in all the procedure rooms of Jefferson Surgical Center Endoscopy, Philadelphia. In addition, Table 5 lists the drugs that are available in the common area. Some of the hospital-attached centers might have access to the hospital pharmacy and might have access to any medication they might need through the pneumatic tube systems. Freestanding centers with no access to the hospital via a bridge or tunnel are not permitted to transfuse blood or blood products, even in an emergency.

Some of the common peri-procedural patient-related concerns/events that need to be addressed are atrial fibrillation, hypertension, renal failure, pacemaker/AICD uncontrolled diabetes, aspiration, bleeding, and perforation. Their peri-procedural management largely depends on the type of center performing the procedure. Hospital-attached centers have more freedom and can transfer these patients (if needed) before or after the procedure with relative ease. Freestanding centers need an ambulance service. There is no evidence to support the cancelation of patients presenting for GI endoscopic procedures solely based on a specific BP measurement or ventricular rate in patients with atrial fibrillation. Such cases should be reviewed on a case-by-case basis [27]. 

Hemorrhage, abdominal pain, and perforation are some of the most common causes of unplanned hospital visits after colonoscopy [28]. Some of the variables associated with unplanned visits are a history of fluid and electrolyte imbalance, psychiatric disorders, the absence of prior arrhythmia, and increasing age past 65 years. Patients with gastrointestinal symptoms have been found to experience a higher rate of polypectomies and have reported higher illicit drug use compared with those with non-gastrointestinal complaints.

## 7. Anesthesia and Sedation Issues

**Figure 2:**
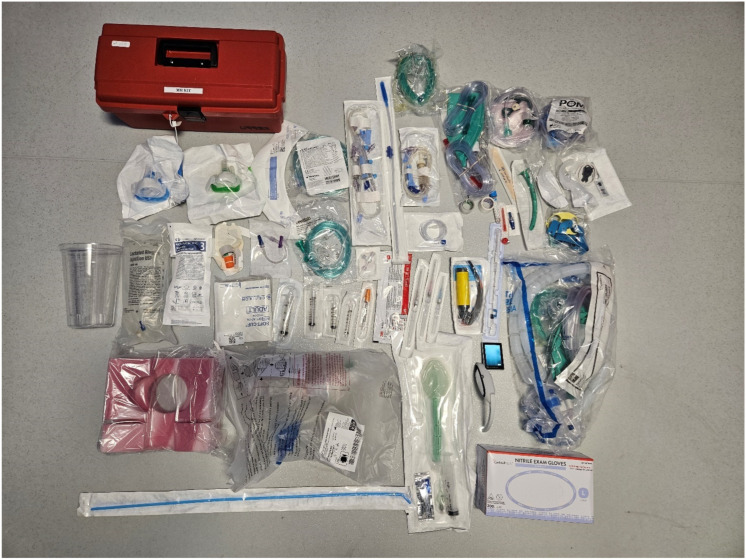
Anesthesia equipment available in all endoscopy rooms.

It is important to have all the necessary drugs and equipment to provide safe sedation. Figure 2 contains all the equipment stocked in endoscopy rooms at the Jefferson Surgical Center Endoscopy, and they are listed in Table 6. In addition, an anesthesia machine should be available. While some centers have an anesthesia machine in every endoscopy suite, some will have a portable anesthesia machine kept in a work room for occasional/emergency needs. To a large extent, it depends on the complexity of the cases performed. At Jefferson Surgical Center Endoscopy, Philadelphia, an anesthesia machine is a component of every endoscopy suite.

**Figure 3:**
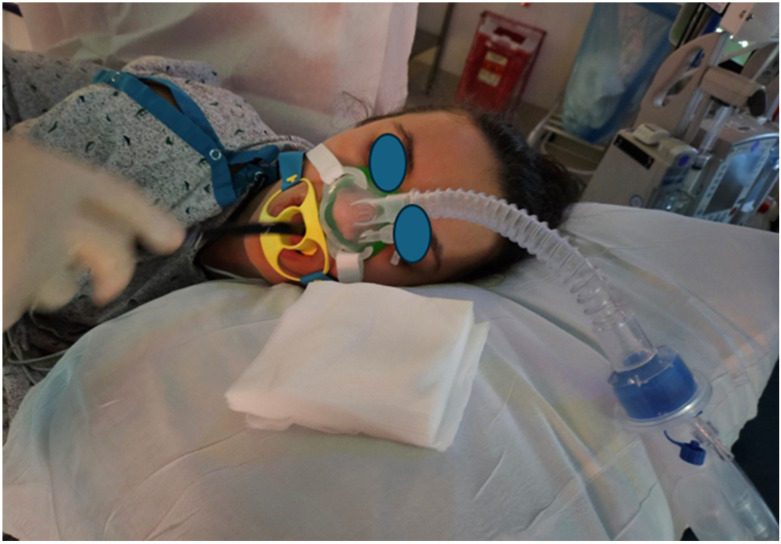
An adult patient undergoing EGD and airway supported with a nasal CPAP device.

**Figure 4:**
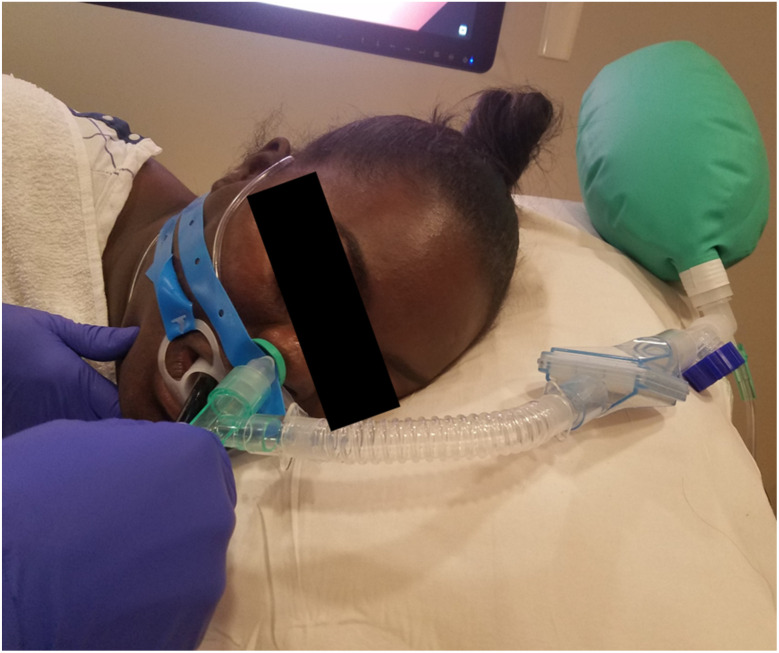
A nasal airway connected to a Mapleson breathing system during an EGD.

Majority of the patients are administered propofol, and the dose is titrated to achieve deep sedation. Patients undergoing endoscopic retrograde cholangiopancreatography (ERCP) are either intubated or provided with deep sedation with appropriate airway modification. Some of the common airway modifications are detailed in Figure 3 and Figure 4. These techniques can preempt desaturation by prolonging apnea time, providing continuous positive airway pressure with apneic insufflation of oxygen, maintaining functional residual capacity, and allowing some degree of positive pressure ventilation. ERCP airway management is still debated, and the practice is center-dependent [29]. The depth of sedation should be titrated with an aim to maintain airway reflex during colonoscopy (so that patient can cough and protect the airway, in the event of passive regurgitation), while during an esophagogastroduodenoscopy, the aim is to suppress the laryngeal reflexes to avoid coughing and laryngospasm. The administration of a short acting opioid such as fentanyl (25–50 microgram increments) can suppress the pharyngeal reflexes and reduce the need for propofol, while propofol alone is sufficient in most colonoscopies. Propofol is typically administered as a bolus followed by an infusion.

Aspiration and hypoxemia are some of the most common adverse events observed in patients undergoing GI endoscopic procedures with deep sedation. In the USA, most of the anesthetics are provided by a certified nurse anesthetist under the supervision of an attending anesthesiologist. It is important to be prepared to address such events. The attending one should be contactable quickly and immediately available in the procedure room if a second pair of hands are needed and decisions are to be made. At Jefferson Surgical Center Endoscopy, every procedure room is equipped with a phone that can speed dial the attending anesthesiologist.

## 8. Planning and Organization: Role of Medical and Nursing Staff

Every staff member working in the endoscopy center plays an important role, and they are expected to function ably and responsibly within their defined position. The responsibility for hiring the appropriate nursing staff and keeping them motivated rests with the nursing director of the unit, who works closely with the medical director, typically an anesthesiologist or a gastroenterologist. This ensures high patient satisfaction along with low cancelation and complication rates. The leaders should be aware of the federal, state, and local regulatory requirements and the staffing needs. Appropriate breaks should be built in the schedule or sufficient staff should exist to provide such breaks. According to the American Nurses Association, almost two-thirds of nurses (62%) experience burnout. WHO defines burnout as mental and physical exhaustion, mental distance from the job, cynicism about the job, and reduced efficacy in the workplace [30,31,32].

Often, nurses multitask wherein they substitute for a technician or another role. With propofol gaining popularity, both among gastroenterologists and patients, the role of moderate sedation has diminished. As a result, the skills of many nurses who are trained to administer moderate sedation and in advanced life support techniques have become redundant, and some of the nurses find it frustrating. Agrawal et al. collected responses from 65 endoscopy units and found that registered nurses did not perform tasks commensurate with their education and training [33]. In their study, they found that about half of all endoscopy units performed all procedures with propofol sedation, while a quarter used propofol for 75–99% of their procedures. These figures were published in 2018, and the trend has shifted further towards deep sedation, mainly propofol. As a result, the role of nurses in the GI endoscopy suite would continue to be one of assisting the endoscopist in various capacities including a technician.

The staffing arrangement on any given day at Jefferson Surgical Center Endoscopy (in Philadelphia) is variable. The nurses are typically assigned (usually on a rotation basis) to various locations such as the preprocedure bay, procedure rooms, and recovery bay. Daily staffing needs depend on the number of beds, procedure rooms in operation, and the patient volume. It is important to avoid large fluctuations in case load and unnecessary case cancelations/rescheduling/transfers. Such occurrences can bring down staff morale and increase avoidable frustration. 

Among the various compliance adherences needed for the successful running of an endoscopy center, the time-out process (TOP) is considered one of the most vital components for patient safety. Some of the identifiable barriers to TOP compliance include a lack of designated team members to lead TOP, inconsistent documentation of TOP, irrelevant safety checklist items not applicable to endoscopic procedures, and lack of patient safety culture. By understanding and actioning on these items, Kara Raphael et al. (2019) increased their TOP compliance to 95.3% from 69.6% [34].

As payers such as Medicare are encouraging physicians to perform more procedures in ASCs (to reduce costs) and add new procedures in their coverage, it is important to avoid violations and complaints. It is observed that infection control and the quality of care and treatment are recurring themes in failed compliances during inspections [35,36]. In a study, 77% of ambulatory surgery centers had at least one violation, and 25% of them had serious deficiencies. During the period 2013–2017, infection control issues were the most frequently cited type of deficiency, making up about one-fifth of all violations. It is vital that all anesthesia medications are capped, labeled, and always stored appropriately. In this study, fortunately, there were fewer patient complaints in the same period compared to previous ones.

Some of the other common ambulatory surgery center violations (as per the report of fire code inspections conducted by the state fire marshal’s office of Olympia, Washington state) are as follows: not conducting and documenting the 30 s monthly test and the 90 min annual test of the emergency lighting, sharing a waiting room with a clinic, the absence of annual maintenance, failure to replace batteries every five years, the absence of timely maintenance, failure to conduct fire drills, the absence of weekly, monthly, and annual generator inspection and testing, the use of extension cords, unapproved multi-plug adapters, exposed wiring, and junction boxes without covers, the presence of penetrations in the fire-rated construction, either the absence of an evacuation and relocation plan or failure to update the same, poor maintenance of fire extinguishers, and having alcohol-based dispensers within one inch of outlets, light switches, and other electrical equipment [37].

## 9. Discharge

### 9.1. Criteria

At Jefferson Surgical Center Endoscopy, Philadelphia, one of the largest inner city ambulatory endoscopy centers, patients are monitored for at least 30 min. Aldrete criteria is used while evaluating for readiness to discharge [38]. The scoring is based on five parameters—respiration, circulation, consciousness, color, and level of activity. The patients score should be 10 (maximum) or the score at the time of admission. In addition, variables such as pain, nausea/vomiting, and ability to drink are assessed, documented, and considered for discharge.

### 9.2. Post-Discharge Follow-Up, Patient Satisfaction

Some hospitals routinely make a phone call the day after the endoscopy procedure to gather information on patient satisfaction and experience. The selective use of such a strategy in patients who underwent Roux-en-Y-gastric bypass or sleeve gastrectomy reduced non-urgent hospital returns [39]. Such phone calls also increased patient satisfaction [40]. Other studies have shown no benefits of post-discharge phone calls [41]. Nevertheless, it is important to keep in mind that the phone conversation or other contact one might have with patients with post-discharge complaints may carry legal liability [42]. Unless all patient concerns are appropriately relayed to the involved physician, the liability could fall on the nurse making the phone call, at least partially.

## 10. Avoiding and Managing Cancellations

If a unit is well managed, any need for cancelation will be negligible. A good management should have a well-focused and thorough screening protocol, followed by a mandatory phone call from one of the nurses about 7 days ahead of the procedure to explain matters such as timely cessation of some medications (e.g., blood thinners, SGLT1 Inhibitors and GLP-1 agonists) and bowel preparation for colonoscopy. Active inflammation in patients with Crohn’s and diverticulitis should be brought to the attention of the corresponding endoscopist. The risk for perforation is higher in such patients [43]. Any recent MI, Stroke (considering patients’ schedule for a colonoscopy 6–18 months in advance) should call for cancelation and rescheduling. 

One of the commonest causes for a colonoscopy cancelation is poor bowel preparation. The adequacy of preparation determines both the diagnostic and therapeutic yield of a colonoscopy [44]. In this retrospective analysis of 1500 consecutive patients who had a diagnostic colonoscopy as an inpatient at a tertiary level hospital over a 2-year period, 194 (18.8%) patients had colonoscopy cancelations, and 268 (26.0%) had poor bowel preparations. The main factors associated with cancelations were education, ethnicity, and hemoglobin level < 10 g/dL. Factors associated with poor bowel preparation were dementia, gastroparesis, and inpatient opioid use. Academic centers tend to have higher cancelation rates. 

Despite all efforts, occasional challenges are inevitable. It is useful to have a back-up plan to deal with such events. As an example, if the center has a body mass index limitation built into the guidelines, and an occasional patient arrives with height greater than the appropriate BMI, either exceptions should be made (e.g., in the absence of other co-morbidities such as severe sleep apnea, difficult airway) or the patient transferred to the hospital (if the endoscopy center is attached to a hospital). A similar approach is needed for other reasons such as a higher than recommended blood sugar, A1c, etc. 

## 11. Conclusions

We have attempted to present the complexities involved in opening and running a freestanding endoscopy surgical center. A variety of factors such as population demographics, access to other tertiary health care facilities for emergency transfer, disease prevalence, and affordability should be considered. Due to the cost involved in the construction and maintenance, a private equity partnership is an attractive option. Compliance with appropriate regulatory authorities’ requirements is mandatory. 

## Figures and Tables

**Table 1:** Direct access colonoscopy and procedure screening form, Jefferson Surgical Center Endoscopy, Philadelphia, PA, USA.

Non-Electronic Medical Record Referral		
Name: ___________________________________________Date: ______________________ Medications: ____________________________________________________________________ Height: _________Weight: _________BMI: _________PMH: __________		
1. COLONOSCOPY ONLY—Needs Delay or Consultation (please put yes/y or no/n in the space before each diagnosis)		
A. Does the patient have an active GI condition requiring initial GI consult? ___Inherited colon cancer syndrome (Lynch, etc.) ___Cirrhosis ___Crohn’s disease or ulcerative colitis ___Age > 75		A If yes to any, schedule GI consult/office visit before scheduling colonoscopy
B. Postpone/offer consult if urgent request ___Pregnancy ___Patient has myocardial infarct (Heart attack) < 12 months ___PT had hospitalization, surgery, joint rep. or vascular grafts < 6 months ___Patient has had a stroke < 6 months ___Treatment for diverticulitis in past 6 weeks		B Schedule after exclusion period or office visit if still having pain of if urgent need for procedure
2. ALL PROCEDURES (that have not been seen in the last year)—for registered nurse (RN) Review or Pre-Admission Testing (PAT) (please place yes/y or no/n in the space before each diagnosis)		
Review patient condition for appropriateness or concerns A ___PT has active angina and/or uses supplemental oxygen ___Cannot walk 2 blocks without stopping due to cardiopulmonary disease ___Not able to lie flat for 2 h due to cardiopulmonary ___New seizure < 6 months B ___Take insulin or blood thinner (anticoagulant) or antiplatelet medication other than aspirin ___Within 6 weeks: new AICD/defibrillator, new pacemaker, or new diagnosis atrial fibrillation ___Within 2 months: hospitalized, started a new medicine, or increased the dose of a medicine for asthma, COPD, or sleep apnea ___Within 3 months: heart surgery (6 weeks for AICD/defibrillator or pacer) or heart catheterization ___Within 3 months: new or worsening angina, pneumonia, or COVID ___Severe heart valve problem (which has not yet been fixed/replaced) ___Dialysis or CKD/advanced kidney disease (GFR < 15) ___History of LVAD, cardiac arrest, cirrhosis, or severe liver disease ___Hospitalized, started a new medicine, or increased the dose of a medicine within 2 months for treatment of asthma, COPD, or sleep apnea ___History of transplant: heart, lung, kidney, bone marrow, small intestine ___Personal or family history of serious problem or complication related to anesthesia ___History of head, throat, or neck cancer or radiation, or history of tracheostomy ___History of a bleeding disorder (Von Willebrand, hemophilia, other) ___History of pheochromocytoma (endocrine tumor) NOT previously cured with surgery ___History of cystic fibrosis or oxygen use at home ___Pregnant		Schedule procedure and send to PAT
3. ALL PROCEDURES (that have not been seen in the last year)—Special Considerations		
Do you have concern about patients’ ability to follow prep instructions or comply? Ability to understand English Low health literacy Is a paraplegic or quadriplegic Ability to read or understand instructions Numerous cancelations or poor prep in past Low socio-economic status		ㅁ Schedule ㅁ Notify navigator
Does the patient have a history of ANY of the following:		
1. Kidney disease (any of these is a YES) GFR < 30 Kidney insufficiency OR failure OR on dialysis Need, or had, a kidney transplant Kidney problems OTHER THAN stones, cancer, cysts	Yes to ANY of these	Use 4 L (ex. CoLyte^®^) PEG-ELS a. Not high risk for inadequate prep 4 L: 2 L 5 pm, 2 L 5 h before colon 2 L: 1 L 5 pm, 1 L 4 h before colon b. High risk for inadequate prep 4 L: 2 L 3 pm, 2 L 6 pm, 2 L 5 h before colon 2 L: 1 L 3 pm, 1 L 6 pm, 1 L 4 h before colon
2. Heart disease (any of these is a YES) Congestive heart failure or CHF Need, or had, a heart transplant Has a defibrillator Arrhythmia or abnormal heart rhythm	Yes to ANY of these	See above
3. Liver disease Cirrhosis OR on list for liver transplant	Yes to ANY of these	See above
4. Hyponatremia OR low sodium concentration in blood	Yes to ANY of these	See above
5. Other condition as determined by CRNP/PA/Nurse/Physician warranting PEG-ELS	Yes to ANY of these	See above
Is patient high risk for inadequate prep? • History of inadequate colon prep • Any use of opioids or tramadol • BMI > 35 • Bowel movement < 3 x/week • Any use of tricyclic antidepressant • History of stroke • On diabetes medication > 5 years	Yes to ANY of these	High dose Miralax-Gatorade (PEG-SD): (diabetics use Gatorade Zero with no sugar) • Bisacodyl at 3 pm • PEG-SD 2 L 3 pm, 2 L 6 pm, and 2 L 5 h before colonoscopy
If patient is NOT a high risk for inadequate prep	-->	Standard dose Miralax-Gatorade (PEG-SD): (diabetics use Gatorade Zero with no sugar) • Bisacodyl at 3 pm • PEG-SD 3 doses: 1 L 4 pm, 1 L 7 pm, and 1 L 4 h before colonoscopy

**Table 2:** List of procedures considered appropriate at Jefferson Surgical Center Endoscopy, Philadelphia, PA, USA.

Esophagogastroduodenoscopy/Enteroscopy;
Colonoscopy;
Flexible Sigmoidoscopy;
Ileoscopy;
Endoscopic Retrograde Cholangiopancreatogrphy;
Endoscopic Ultrasound with fine needle biopsy and aspiration;
Rectal Ultrasound;
Endoscopic dilation of strictures and senosis using balloons, savary dilators, and bougenage dilatos;
Thermocoagulation of bleeding sourcesin esophagus, stomach, small intestine, colon, and rectum (including argon plasma coagulation);
Thermoablation therapy for the disintegration of malignant and pre-malignant tissue (including RFA and cryo ablation);
Percutaneous endoscopic gastrostomy tube placement;
Local contact and infiltration anesthesia;
Esophageal, gastric, intestinal, and rectal manometry;
24 h pH monitoring;
Endoscopic placement of stents in esophagus, stomach, small intestine, colon, and rectum (including argon plasma coagulation);
Endoscopic suturing;
Capsule colonoscopy;
Capsule endoscopy;
Revision of prior gastric bypass using endoscopic suturing device;
Gastric balloon;Endoscopic mucosal resection (physician determines if appropriate for HC)—added 6/3.

**Table 3:** (**A**) Inclusion criteria for patient appropriateness from the anesthesiology stand point adapted at Jefferson Surgical Center Endoscopy, Philadelphia, PA, USA. (**B**) Exclusion criteria for patient appropriateness from the anesthesiology stand point adapted at Jefferson Surgical Center Endoscopy, Philadelphia, PA, USA.

(**A**)
Physical status Considerations
ASA Class I–III (ASA III is at the discretion of medical director)
○ ASA I—A normal, healthy patient
○ ASA II—A patient with mild systemic disease
○ ASA III—A patient with a severe systemic disease that limits activity but is not incapacitating
Social Considerations
Patient provides consent for surgery in an ambulatory setting and accepts associated risks
Patient must have a ride home and care by an adult
○ Public transit, taxis, or ride-share service use are prohibited at discharge
Patient or caregiver must be competent and understand post-operative instructions
Patient home environment should be suitable for post-operative care
Surgical Considerations
Surgical procedure is elective and outpatient with post-operative admission/transfer not anticipated
Surgical blood loss requiring transfusion is not anticipated
Involvement in major blood vessel not anticipated for procedure
All surgical equipment for type of procedure is on site
No invasive monitoring (pressure transduction, TEE) required for the procedure
Medical Considerations
BMI less than 50; BMI > 50 at the discretion of medical director
Post-operative mechanical ventilation is not anticipated
Post-operative pain anticipated to be controlled primarily with oral analgesia, regional, or local anesthesia
(**B**)
Patients with:
○ Substance Use Disorder
▪ Acutely intoxicated patient, caregiver, or guardian (including cannabinoids) on DOS
○ Severe sleep apnea
▪ AHI ≥ 30 (adult) or ≥ 10 (pediatric)
▪ SpO_2_ nadir ≤ 80% (adult) or ≤75% (pediatric)
▪ Severe as otherwise indicated on sleep study or suspected based on history/exam
▪ discussion with surgical team if their surgical procedure is for study or intervention
○ History of difficult airway management
▪ Case by case basis to be evaluated prior to day of surgery to determine severity
○ Known personal or family history of malignant hyperthermia, or associated conditions
▪ Central core disease
▪ Multiminicore disease
▪ King-Denborough syndrome
○ Uncontrolled diabetes
▪ Glucose ≥ 300 mg/dL on day of surgery
▪ Patients requiring an insulin infusion (not patients with own pumps)
○ Psychological instability or agitated requiring a 1:1 companion
○ Stroke or TIA within
▪ 3 months (non-elective procedure)
▪ 9 months (elective procedure)
○ Uncontrolled hypertension
▪ SBP ≥ 210 mmHg
▪ DBP ≥ 110 mmHg
○ Myocardial infarction within
▪ <3 months
▪ 3–6 months and need to stop DAPT
○ Unstable angina
○ Severe valvular disease
▪ Echocardiogram within prior year required for patients with
• Moderate or severe valvular disease
• Change in clinical status or physical exam since last evaluation
○ Severe pulmonary hypertension
○ Congestive heart failure on study in last six months
▪ LVEF ≤ 30%
▪ Moderate to severely reduced RV function
▪ Severe LV diastolic dysfunction
○ Cardiac Implantable Electronic Device (CIED)
▪ Interrogation report within past six months not available
▪ Unable to obtain information about
• CIED type
• CIED-dependent pacing
• CIED function
▪ Reprogramming anticipated
• e.g., monopolar electrocautery or radiofrequency ablation above umbilicus, surgery site within 15 cm of CIED
○ End Stage Renal Disease—Stage 4 (GFR < 30 mL/min)
▪ Require Potassium draw within 24 h
▪ Dialysis Patients
• Schedule procedure for day after dialysis
• Require BMP morning of procedure; preferably patient to arrive at lab 2 h prior to procedure
○ Sickle cell anemia with crisis
○ Hematologic and bleeding disorders
○ Severe or disabling neuromuscular disorder
▪ Symptoms (including but not limited to)
• Autonomic dysfunction
• Bulbar muscle weakness
• Comorbid cardiopulmonary disease (e.g., restrictive lung disease, cardiomyopathy)
▪ Myasthenia gravis and any of the following
• Modified Osserman and Genkins classification grade ≥ 2
• Anti-acetylcholine antibody positivity
• Presence of thymoma
• Chronic comorbid pulmonary disease
• Vital capacity < 2.9 L
• Prior episode of respiratory failure/myasthenia crisis
○ Active multiple sclerosis
▪ With concern for difficulty with extubation
▪ Evaluate prior to day of surgery to determine severity
○ Newly diagnosed systemic disease that is poorly compensated or incompletely evaluated
▪ All chronic and acute conditions should be appropriately optimized prior to day of surgery

**Table 4:** Drugs stocked in each endoscopy room at Jefferson Surgical Center Endoscopy, Philadelphia.

Anesthetics/Sedatives/Analgesics
Propofol (DIPRIVAN) 1000 mg (100 mL); 7 bottles
Propofol (DIPRIVAN) 200 mg (20 mL); 18 vials
Dexmedetomidine (PRECEDEX) 200 mcg (50 mL); 5 vials
Etomidate (AMIDATE) 40 mg (20 mL); 4 vials
Fentanyl (SUBLIMAZE) 100 mcg (2 mL) injection; 7 vials
Isoflurane (FORANE) (100 mL); 1 bottle
Ketamine (KETALAR) 10 mg/1 mL (20 mL); 4 vials
Ketorolac (TORADOL) 15 mg (1 mL); 6 vials
Midazolam 2 mg (2 mL); 10 vials
Cardiac/Vasopressors/Inotropes
Adenosine (ADENOCARD) 6 mg (2 mL); 3 vials
Amiodarone (CORDARONE) 150 mg (3 mL); 4 vials
Atropine (ATROPINE) 0.1 mg/1 mL (10 mL); 2 syringes
Calcium Chloride (CALCIUM CHLORIDE) (10 mL); 2 syringes
Ephedrine (EPHEDRINE) 50 mg (5 mL) syringe
Epinephrine 0.1 mg/mL (LUER-LOCK) 1 mg (10 mL); 10 syringe s
Esmolol (BREVIBLOC) 100 mg (10 mL); 4 vials
Glycopyrrolate (ROBINUL) 0.2 mg (1 mL); 22 vials
Labetalol (TRANDATE) 20 mg (4 mL); 8 syringes
Metoprolol (LOPRESSOR) 5 mg (5 mL); 6 vials
Nicardipine (CARDENE) 25 mg (10 mL); 2 vials
Norepinephrine (LEVOPHED) 4 mg (4 mL); 3 vials
Phenylephrine—(NEO-SYNEPHRINE/VAZCULEP) 100 mcg/1 mL (10 mL); 9 syringes
Phenylephrine in sodium chloride 0.9% (VAZCULEP) 50 mg (250 mL); 1 bag
Sedative/anticholinergic/antiemetic
Diphenhydramine (BENADRYL) 50 mg (1 mL); 5 vials
Ondansetron (ZOFRAN) 4 mg (2 mL; 16 vials
Bronchodilator
Albuterol HFA (PROVENTIL, VENTOLIN) 90 mcg inhaler; 1 canister
Antibiotics
Cefazolin (ANCEF) 1 g; 14 vials
Vancomycin (VANCOCIN) 1 g; 2 vials
Diuretics
Furosemide (LASIX) 20 mg (2 mL); 5 vials
Miscellaneous
Dexamethasone
Dextrose 50%
Glucagon (GLUCAGON) 1 mg; 2 vials
Heparin sodium (HEPARIN) 1000 units/1 mL (10 mL); 8 vials
Hydrocortisone sod succinate (SOLU-CORTEF) 100 mg (2 mL); 3 vials
Lidocaine 2%, Pre-Filled, (XYLOCAINE) 20 mg/1 mL (5 mL); 3 vials
Lidocaine, Pre-Filled (XYLOCAINE) 100 mg (5 mL); 3 syringes
Naloxone (NARCAN) 0.4 mg (1 mL); 4 vials
Neostigmine methyl sulfate (PROSTIGMINE) 1 mg/1 mL (3 mL); 5 syringes
Sodium bicarbonate 8.4% 50 mEq (50 mL); 1 syringe
Skeletal Muscle Relaxants/Reversals
Rocuronium 50 mg (5 mL); 6 vials
Succinylcholine chloride 200 mg (10 mL), 10 syringes
Sugammadex (BRIDION) 100 mg/1 mL (2 mL); 10 vials
Neostigmine methyl sulfate (PROSTIGMINE) 1 mg/1 mL (3 mL); 5 syringes

**Table 5:** Medications stored in a common area Pyxis at Jefferson Surgical Center Endoscopy, Philadelphia.

Analgesics/Anti-inflammatory/Sedatives/Anesthetics/Cardiac
Acetaminophen (1000 mg injections and Tablets)
Alprazolam (XANAX) 0.5 mg tablets
Aspirin chewable (BABY ASPIRIN) 81 mg tablet
Clonazepam (KLONOPIN) 0.5 mg tablets
Hydromorphone (DILAUDID) 2 mg (1 mL) injections
Indomethacin (INDOCIN) 50 mg suppositories
Ketamine (KETALAR) 50 mg/1 mL (10 mL) vials
Lidocaine PF 2% (XYLOCAINE) (2 mL) vial
Lorazepam inj (ATIVAN) 2 mg (1 mL) vials
Meperidine (DEMEROL) 25 mg (1 mL) syringes
Morphine 4 mg (1 mL) syringes
Nitroglycerin (NITROSTAT) 0.4 mg tablets
Nitroglycerin 100 mcg/1 mL (5 mL) vials
Nitroglycerin 2% ointment (NITRO-BID) 1 g packet
Oxycodone IR (ROXICODONE IR) 5 mg tablets
Vasopressin (VASOSTRICT) 20 units (1 mL) vial
Bronchodilators
Albuterol 0.083% (VENTOLIN) 2.5 mg (3 mL), for nebulizer
Antibiotics
Bacitracin ointment 500 unit 14 g tubes
Cefazolin (ANCEF) 1 g vials
Ciprofloxacin (CIPRO) 400 mg (200 mL) bags
Gentamicin (GARAMYCIN) 40 mg/1 mL (2 mL) vials
Gentamicin (GARAMYCIN) 80 mg (50 mL) bag
Metronidazole in sodium chloride 0.9% (FLAGYL) 500 mg (100 mL) bags
Vancomycin (VANCOCIN) 1 g vials
Muscle relaxants
Cisatracurium 20 mg (10 mL) vials
Antiemetics
Metoclopramide (REGLAN) 10 mg (2 mL) vials
Promethazine (PHENERGAN) 25 mg (1 mL) vial
Scopolamine 1 mg-over-3 days (TRANSDERM SCOP) patch
Miscellaneous
Dextrose 50% 25 g (50 mL) syringes
Glucagon 1 mg vials
Heparin flush PF (HEP-LOCK) 500 units (5 mL) syringes
Insulin human regular 100 units/mL (Humulin R) units
Insulin lispro 100 units/mL (Humalog) units
Insulin regular in sodium chloride 0.9% (Humulin R Infusion) 100 unit (100 mL) bags
Iohexol (OMNIPAQUE) 350 mg/1 mL (100 mL) bottles
Magnesium sulfate 20 g (500 mL) bag
Methylprednisolone PF (SOLU-MEDROL) 40 mg/1 mL injections
Naloxone (NARCAN) 0.4 mg (1 mL) vials
Onabotulinumtoxina (BOTOX A) 100 units vial
Oxymetazoline 0.05% nasal (AFRIN) (15 mL) spray bottle
Pantoprazole (PROTONIX) 40 mg vial
Sodium bicarbonate 8.4% 50 mEq (50 mL) vial
Sodium chloride 0.9% (sodium chloride 0.9% (1000 mL) bag) (1000 mL) bags
Triamcinolone acetonide (KENALOG-10) 50 mg (5 mL) vial

**Table 6:** List of equipment in the endoscopy suite at Jefferson Endoscopy Center, Philadelphia.

Malignant Hyperthermia Kit (one per unit)
Nasal CPAP masks
Endotracheal Tubes (various sizes)
Nasal canula/oxygen tubing with CO_2_ sample line
IV infusion tubing
Face Mask
Mapleson breathing system.
AMBU bag (in each procedure room)
Oxygen mask
Procedural Oxygen Mask (POM)
Disposable pulse oximeter probes
Bite Blocks (various sizes)
Oropharyngeal airways (various sizes)
Nasopharyngeal airways (various sizes)
Tongue depressor
Intravenous cannula caps
Needles (various sizes)
Vacutainers
Tapes (both synthetic and hypoallergenic/micropore)
IV extension tubing
Laryngoscope including with video.
Arterial cannula
IV cannulas of different sizes
EKG electrodes
Syringes (various sizes including tuberculin syringe)
BP cuffs (various sizes)
Short IV extension tubing (that connects to the IV cannula)
IV kit (tagaderm, tourniquet, sterile alcohol wipes, and tape)
Laryngoscope blades
IV fluids
Suction cannisters.
Disposable pillows
IV fluids (saline)
Bougie (to assist with difficult intubations)
Laryngeal Mask Airways
Nitrile examination gloves
Anesthesia Machine breathing circuit
